# Sensory Trial of Quintuple Fortified Salt—Salt Fortified With Iodine, Iron, Folic Acid, Vitamin B_12_, and Zinc—Among Consumers in New Delhi, India

**DOI:** 10.1177/03795721221078361

**Published:** 2022-05-08

**Authors:** Seema Puri, Tejmeet Kaur Rekhi, Tinku Thomas, Meena Haribhau Jadhav, Venkatesh Mannar, Levente L. Diosady

**Affiliations:** 1 Department of Food and Nutrition, Institute of Home Economics, University of Delhi, New Delhi, India; 2 St John’s Research Institute, Bangalore, Karnataka, India; 3 Nutrition Impact Solutions Inc., North York, Ontario, Canada; 4 The Micronutrient Initiative, Ottawa, Ontario, Canada; 5 Department of Chemical Engineering and Applied Chemistry, University of Toronto, Toronto, Ontario, Canada

**Keywords:** multiple fortified salt, fortification, micronutrients, sensory trial

## Abstract

**Background:**

Micronutrient deficiencies are a cause of significant public health burden and loss of gross domestic product, especially in developing countries. Multiple fortified salt can potentially address this challenge at scale and in a cost-effective manner.

**Objective:**

This laboratory-based sensory trial evaluated the acceptability of quintuple fortified salt (Q_5_FS), that is, iodized salt (IS) fortified with additional 4 micronutrients: iron, folic acid, vitamin B_12_, and zinc. Iodized salt and double fortified salt (DFS), that is, IS fortified with iron, are used for comparison.

**Methods:**

Forty-five respondents were recruited by open invitations to the university staff and their families. Each study participant rated 10 food items each in a set of 3 identical preparations differing only in the salt used. A 5-point hedonic scale was used to rate each dish on 6 sensory attributes: appearance, color, aroma, taste, texture, and aftertaste. Finally, the dish was rated on the attribute of overall acceptability—a subjective combined score based on all sensory attributes considered together.

**Results:**

Among the 3 salt types, there was no difference in scores for the sensory attributes of appearance, aroma, taste, texture, and aftertaste, and the attribute of overall acceptability. Color in IS scored significantly higher than in Q_5_FS and DFS, but there was no difference between the scores of DFS and Q_5_FS.

**Conclusions:**

The 3 salts IS, DFS, and Q_5_FS are comparable to each other in all sensory properties, except for color. This study concludes that Q_5_FS is organoleptically acceptable under ideal conditions.

## Introduction

Food fortification is widely accepted as a cost-effective strategy to reduce micronutrient deficiencies (MNDs) of public health significance, especially in the context of low-income countries. The impact potential of food fortification can be further enhanced by multiple fortification, that is, adding multiple nutrients to a single food vehicle. Micronutrient deficiencies affect more than 2 billion people globally,^1^ leading to mental impairment, poor health, low productivity, and even death.^2^ Iodine, iron, vitamin A, folic acid, and zinc have the highest public health significance globally^3^ either due to the widespread prevalence of their deficiencies or their devastating effect on a specific population group. Salt iodization has significantly reduced the estimated 2 billion individuals who had insufficient iodine intake and an estimated 38 million newborn babies who were at risk of iodine deficiency,^4^ and the estimated 19 million at risk of irreversible brain damage and reduced cognitive function.^5^ Iron deficiency is the most common cause of anemia that affects 43% of children younger than 5 years and 38% of pregnant women globally.^6^ The global prevalence of neural tube defects (NTDs) is estimated at around 18.6/10 000 live births, with an estimated 260 100 NTDs in the year 2015.^7^ A majority of the NTDs were found to be prevented with folic acid supplementation during pregnancy,^8^ indicating that folic acid deficiency is a major cause of NTDs. Vitamin B_12_ deficiency is a contributing factor to folic acid deficiency and anemia.^8^ Zinc deficiency is found to be an important contributing factor to morbidity and mortality, especially in children. An estimated 2 billion of the global population could be zinc deficient.^9^ Addressing MNDs can not only contribute significantly to the reduction of the global disease burden but also remove barriers to productivity, economic growth, and poverty eradication.

Micronutrient deficiencies transcend generations and often coexist in populations vulnerable due to a combination of factors. The immediate causes of MNDs also include reduced absorption due to infection, disease, and inflammation; however, reduced intake of these micronutrients remains a major cause.^3^ Addressing multiple MNDs together is a public health imperative, and food fortification is a proven, sustainable, and cost-effective strategy with the potential to reach large populations simultaneously. However, policy decisions of whether to adopt food fortification as a public health approach, the choice of food vehicle, the micronutrients to include, need to be informed by epidemiological context, safety concerns, optimal use of resources, and ethical trade-offs.^10^

Multiple fortification has been adopted in wheat flour and rice; however, salt has the unique advantage of being a universally, uniformly, and consistently used commodity, irrespective of socioeconomic status. It is also centrally processed, and therefore, amenable to large-scale fortification. In the past, formulations of double fortified salt (DFS; iodine + iron) have been tested, and the acceptability and efficacy trials for these formulations have shown promising results.^11-13^ More recently, the University of Toronto has developed quintuple fortified salt (Q_5_FS) with additional micronutrients (folic acid + vitamin B_12_ + zinc) along with iron and iodine, as a potentially viable strategy to alleviate MNDs. However, it is essential to evaluate the sensory acceptability of these formulations as a precursor to efficacy trials and before wider application. We realize the possibility of duplication of interventions in a country such as India, where additional fortification of multiple fortified vehicles is layered on ongoing pharmacological supplementation programs.^10^ However, we foresee food fortification as a sustainable, cost-effective strategy, especially in populations where pharmacological supplementation has had historically low coverage. The food habits in a culturally diverse population of India make it difficult for any single food vehicle to achieve the status of a public health product with universal consumption, except for certain condiments like salt and oil. The success of any public health approach depends both on its efficacy and implementation simplicity. The sources of the staples wheat and rice, currently focused for multiple fortification, are varied and it may be logistically unfeasible to achieve high levels of sustained coverage with such food vehicles.^10^ Salt fortification transcends regional differences in dietary patterns, gender discrimination in food allocation, has a potential for global application, presents a readily available fortification platform established by universal salt iodization, and involves a fortification vehicle with mandatory legislations in most countries globally. Multiple fortification and its successful field application, therefore, have advantages unique to salt.

This study is a laboratory-based sensory trial for measuring and comparing the acceptability score of 3 formulations of fortified salt: (1) Q_5_FS, that is, fortifying table salt with 5 nutrients: iodine, iron, folic acid, and vitamin B_12_, and zinc; (2) DFS, that is, table salt fortified with iodine and iron; and (3) iodized salt (IS). The purpose of this study is to evaluate the sensory acceptability of Q_5_FS formulation used for preparing commonly consumed dishes, using DFS and IS for comparison.

The Q_5_FS and DFS developed by the University of Toronto use the microencapsulation technology to coat and protect the micronutrient/s premix particle and create a barrier of separation from the iodine present in the table salt. Quintuple fortified salt is developed to be essentially identical to IS in appearance and taste. Even with white coating, the premix particles are visible in raw salt both in Q_5_FS and DFS; but no change in taste or texture of cooked food is expected. The coating dissolves during the process of food preparation when subject to moisture and heat, releasing the micronutrients in food, causing minor color changes (often imperceptible) in the food prepared. Except for a mild color change in cooked food (due to the presence of iron in Q_5_FS), the food is expected to be similar to that cooked with IS. The composition of the different salt formulations based on the number of micronutrients added to IS is presented in Table 1.

**Table 1.** Micronutrient Profile of Fortified Salts.

Ingredients	US NIH RDAs Micronutrients	Quintuple fortified salt^a^		Double fortified salt^a^		Iodized salt^b^	
		Micronutrients/10 g of fortified salt	Micronutrients % RDA/10 g salt	Micronutrients 10g of fortified salt	Micronutrients % RDA/10 g salt	Micronutrients/10 g of fortified salt	Micronutrients % RDA/10 g of salt
Sodium chloride							
Zinc	11 mg	3.52 mg	32.0%				
Iron	18 mg	9.20 mg	51.1%	10 mg	56%		
Iodine	150 µg	150 µg^c^	100%	150 µg^c^	100%	150 µg^c^	100%
Folic acid	400 µg (DFE)	250 µg FA (416 DFE)	104%				
Vitamin B_12_	2.4 µg	2.49 µg	103%				

Abbreviations: DFE, Dietary Folate Equivalent; RDAs, Recommended Dietary Allowances; US NIH, United States National Institutes of Health.

^a^ Data source: University of Toronto.

^b^ Data source: Food Safety and Standards Authority of India.

^c^ Adjusted for expected value at the consumer level.

## Methods

### Study Settings and Population

The study was conducted at the campus of the Institute of Home Economics, a teaching and research institute under Delhi University in New Delhi, India. Invitations were issued to faculty and staff, and individuals residing around the college campus. A diverse sample of study participants across income groups, occupations, education levels, gender, and age were recruited. The information sheet on the study was shared and explained to the participants; however, they were not trained to evaluate.

### Study Design

#### Preparatory phase

In the preparatory phase, women from rural backgrounds in Bawal district of Haryana state (adjacent to Delhi) were interviewed to identify the commonly consumed food preparations in these households. A list of these food items and methods of preparation were compiled. The data were collected from 26 women above the age of 18, following a qualitative approach, until a saturation point was reached, and no new data were being generated. A semistructured interview guide was used for collecting data on sociodemographic variables, salt consumption patterns, commonly consumed food items, and methods of preparation. The food preparations identified were then tried in laboratory settings at the Department of Food and Nutrition, Institute of Home Economics, New Delhi. The commonly consumed dishes were then standardized in the laboratory, based on the recipes shared by the rural women. Each dish was prepared using standardized procedures twice initially and repeated for a third time in case of a dish showing different results in the first 2 times. The food preparations were evaluated by 4 trained faculty staff and research staff using a 5-point hedonic scale for 6 sensory attributes: appearance, color, aroma, taste, texture, and aftertaste. Additionally, the overall acceptability of the 3 fortified salt formulations was also assessed using the same scale. A total of 50 food items were tried and standardized in this preparatory phase. An acceptability test for a variety of preparations with Q_5_FS during the preparatory phase of the trial indicated that almost all dishes exceeded the defined minimum acceptability score of 3 (taken from the midpoint of the 5-point hedonic scale). Of these different preparations, 10 food items were randomly included in the sensory trial for comparative sensory evaluation between the 3 types of salt.

#### Consumer sensory evaluation

A sensory trial was conducted using Central Location Testing method with prerecruited participants. A sample size of 30 participants is sufficient to observe a difference of 3 units in mean acceptability scores for the different salts, with the standard deviation for the difference in scores between the salts considered as 5 units. This sample size is sufficient for 80% power and 0.025% level of significance (Bonferroni adjusted for 2 comparisons, that is, of DFS and Q_5_FS with the IS). We recruited a sample size of 45 respondents comprising of a mixed group from different socioeconomic sections. Recruitment was done by an open invitation to faculty, staff, security and housekeeping personnel, students, and their family members. Each participant rated the 10 food preparations, each in a set of 3 preparations prepared using the different salts (IS, DFS, and Q_5_FS) to evaluate a total of 30 dishes. A 5-point hedonic scale was used to rate each dish on the mentioned 6 sensory attributes and the attribute of overall acceptability.

Variations resulting from cooking errors were minimized using standard procedures: (1) using identical ingredients and amounts for identical dishes except for the salt; (2) dishes cooked, in similar utensils, at the same time, for an equal duration of cooking; (3) dishes were prepared in one lot before the trial; and (4) all variants were cooked simultaneously by 3 researchers. Dishes with variants were blinded, and each dish was displayed on a separate counter, distant from each other. Labels A, B, and C were randomly assigned to the variant dishes so that for every preparation, the same variant would not have the same name. The researchers were not blinded to the salt as they were familiar with the salt from earlier trials; however, participants evaluating the dishes were blinded to the salt. The evaluators were called in batches of 5. All evaluations were done on the same day. Participants were not allowed to discuss or interact with each other during the evaluation. They were asked to drink a few sips of water between each assessment. The research team was present to clarify any doubts.

### Data Analysis

The dependent variables in the study were scoring for appearance, color, aroma, taste, texture, aftertaste, and the overall acceptability of the cooked dishes. The overall acceptability included in the analysis is a subjective attribute of the 3 salt formulations measured on the hedonic scale and not a composite scoring of the 6 sensory attributes measured. From the consumer point of view, the subjective attribute of the overall acceptability of the salt is more important for determining acceptance and utilization of the product. Consumers may stop using the product based on their dissatisfaction with any one of the 6 sensory attributes evaluated. Therefore, from the consumer point of view, overall acceptability, although subjective, is more important than the composite score. The independent variables were the 3 types of salt: IS, DFS, Q_5_FS; treated as 3 treatment groups. The scores for the 3 treatment groups were treated as repeated measures for each dependent variable evaluated by the same set of 45 study participants.

The distributions of scores for each sensory attribute for the 3 salt types were examined for normality using the Shapiro-Wilk test and Q-Q plot. For the sensory attributes with normally distributed scores, the difference in mean scores was tested using repeated measures analysis of variance (ANOVA). For the sensory attributes with non-normal distribution, Friedman nonparametric test was used to compare the salts. Differences were considered significant when the *P* value was less than .05. Post hoc pairwise-Wilcoxon signed-rank test was performed for the pairwise comparisons if the tests showed statistically significant differences in scores.

### Ethical Approval

Ethical clearance for the study was obtained from the Institutional Ethics Committee of the Institute of Home Economics in its meeting held on October 16, 2017.

## Results

### Descriptive Statistics

Individual ratings of the study participants for 10 sets of 3 identical food preparations prepared using the 3 different salts were obtained using a 5-point hedonic scale. We performed a descriptive analysis to get the mean scores for each of the 10 food items included in the sensory trial for all the 6 sensory attributes and the additional attribute of overall acceptability for each of the 3 salt types (Table 2).

**Table 2.** Descriptive Statistics (Mean, SD) for Comparing Sensory Scores.

Food items	IS	DFS	Q_5_FS
**Appearance**			
*Bajra khichdi*	3.80 (±0.8)	3.40 (±1.0)	3.60 (±0.8)
*Carrot raita*	3.70 (±1.0)	3.90 (±0.8)	4.00 (±0.6)
*Aloo gobhi sabji*	4.10 (± 0.8)	4.10 (±0.6)	3.80 (±0.8)
*Mix veg pakodas*	4.10 (± 0.6)	4.20 (±0.6)	4.30 (±0.7)
*Masoor dal*	3.80 (± 0.9)	3.60 (±0.9)	3.60 (±0.7)
*Jeera rice*	3.60 (±0.8)	3.30 (±0.7)	3.60 (±0.9)
*Baingan bharta*	3.70 (±0.7)	3.70 (±0.9)	3.50 (±0.6)
*Matar paneer*	4.20 (±0.9)	4.00 (±0.8)	4.10 (±0.6)
*Chickpea curry*	4.10 (±0.6)	4.10 (±0.7)	3.80 (±0.8)
*Bathua saag*	3.70 (±0.8)	3.90 (±0.6)	3.80 (±0.7)
**Color**			
*Bajra khichdi*	3.91 (±0.7)	3.20 (±0.9)	3.40 (±0.8)
*Carrot raita*	3.80 (±1.0)	3.80 (±0.8)	4.10 (±0.6)
*Aloo gobhi sabji*	4.10 (±0.7)	4.00 (±0.7)	3.60 (±0.8)
*Mix veg pakodas*	4.00 (±0.6)	4.10 (±0.6)	4.30 (±0.7)
*Masoor dal*	3.80 (±0.8)	3.60 (±0.8)	3.50 (±0.8)
*Jeera rice*	3.70 (±0.9)	3.20 (±0.7)	3.70 (±0.8)
*Baingan bharta*	3.70 (±0.7)	3.70 (±0.9)	3.60 (±0.7)
*Matar paneer*	4.20 (±0.8)	3.90 (±0.8)	4.10 (±0.8)
*Chickpea curry*	4.20 (±0.7)	4.20 (±0.6)	3.80 (±0.7)
*Bathua saag*	3.90 (±0.7)	3.80 (±0.6)	4.00 (±0.7)
**Aroma**			
*Bajra khichdi*	3.82 (±0.7)	3.60 (±0.9)	3.60 (±0.8)
*Carrot raita*	3.80 (±0.9)	3.80 (±0.8)	4.00 (±0.5)
*Aloo gobhi sabji*	3.90 (±0.7)	4.00 (±0.7)	3.60 (±0.7)
*Mix veg pakodas*	4.10 (±0.6)	4.10 (±0.6)	4.20 (±0.6)
*Masoor dal*	3.90 (±0.7)	3.60 (±0.8)	3.70 (±0.7)
*Jeera rice*	3.70 (±0.8)	3.50 (±0.8)	3.50 (±0.8)
*Baingan bharta*	3.60 (±0.6)	3.70 (±0.7)	3.50 (±0.6)
*Matar paneer*	4.00 (±0.8)	3.80 (±0.7)	4.00 (±0.6)
*Chickpea curry*	4.00 (±0.7)	4.10 (±0.7)	3.80 (±0.9)
*Bathua saag*	3.50 (±0.7)	3.60 (±0.7)	3.70 (±0.7)
**Taste**			
*Bajra khichdi*	3.71 (±0.8)	3.70 (±0.8)	3.60 (±0.8)
*Carrot raita*	3.80 (±0.9)	3.80 (±0.9)	3.80 (±0.6)
*Aloo gobhi sabji*	4.00 (±0.8)	4.00 (±0.7)	3.70 (±0.8)
*Mix veg pakodas*	4.10 (±0.7)	4.20 (±0.7)	4.30 (±0.7)
*Masoor dal*	3.80 (±0.8)	3.70 (±0.8)	3.60 (±0.8)
*Jeera rice*	3.40 (±0.8)	3.50 (±0.8)	3.40 (±0.7)
*Baingan bharta*	3.30 (±0.8)	3.60 (±0.8)	3.50 (±0.7)
*Matar paneer*	4.00 (±0.8)	4.00 (±0.8)	4.00 (±0.7)
*Chickpea curry*	3.90 (±0.7)	3.90 (±0.8)	3.40 (±1.0)
*Bathua saag*	3.40 (±0.8)	3.50 (±0.8)	3.60 (±0.7)
**Texture**			
*Bajra khichdi*	3.86 (±0.7)	3.60 (±0.9)	3.60 (±0.7)
*Carrot raita*	3.80 (±0.8)	3.80 (±0.8)	3.90 (±0.5)
*Aloo gobhi sabji*	4.00 (±0.7)	4.00 (±0.7)	3.90 (±0.8)
*Mix veg pakodas*	4.20 (±0.7)	4.30 (±0.6)	4.40 (±0.6)
*Masoor dal*	3.70 (±0.9)	3.60 (±0.8)	3.60 (±0.9)
*Jeera rice*	3.50 (±0.8)	3.40 (±0.9)	3.50 (±0.8)
*Baingan bharta*	3.70 (±0.7)	3.70 (±0.7)	3.80 (±0.7)
*Matar paneer*	4.10 (±0.9)	4.00 (±0.8)	4.20 (±0.6)
*Chickpea curry*	4.10 (±0.7)	4.00 (±0.6)	3.80 (±0.8)
*Bathua saag*	3.60 (±0.7)	3.70 (±0.6)	3.70 (±0.7)
**Aftertaste**			
*Bajra khichdi*	3.80 (±0.9)	3.80 (±0.9)	3.70 (±0.8)
*Carrot raita*	3.80 (±0.9)	3.73 (±0.8)	3.90 (±0.7)
*Aloo gobhi sabji*	4.00 (±0.7)	4.10 (±0.7)	3.80 (±0.7)
*Mix veg pakodas*	4.20 (±0.8)	4.20 (±0.7)	4.30 (±0.6)
*Masoor dal*	3.70 (±0.9)	3.80 (±0.7)	3.80 (±0.9)
*Jeera rice*	3.60 (±0.7)	3.50 (±0.7)	3.60 (±0.8)
*Baingan bharta*	3.40 (±0.8)	3.70 (±0.8)	3.60 (±0.8)
*Matar paneer*	4.10 (±0.8)	4.00 (±0.7)	4.00 (±0.6)
*Chickpea curry*	4.00 (±0.8)	4.00 (±0.8)	3.80 (±0.9)
*Bathua saag*	3.50 (±0.8)	3.60 (±0.6)	3.60 (±0.7)
**Overall**			
*Bajra khichdi*	3.90 (±0.8)	3.70 (±0.8)	3.60 (±0.7)
*Carrot raita*	3.90 (±0.9)	3.80 (±0.8)	4.00 (±0.5)
*Aloo gobhi sabji*	4.10 (±0.7)	4.10 (±0.6)	3.80 (±0.6)
*Mix veg pakodas*	4.10 (±0.7)	4.20 (±0.6)	4.30 (±0.6)
*Masoor dal*	3.80 (±0.9)	3.80 (±0.8)	3.70 (±0.8)
*Jeera rice*	3.60 (±0.8)	3.50 (±0.7)	3.60 (±0.9)
*Baingan bharta*	3.60 (±0.7)	3.70 (±0.8)	3.60 (±0.7)
*Matar paneer*	4.10 (±0.7)	4.00 (±0.8)	4.10 (±0.6)
*Chickpea curry*	4.10 (±0.6)	3.90 (±0.8)	3.80 (±0.9)
*Bathua saag*	3.60 (±0.7)	3.70 (±0.6)	3.70 (±0.6)

Abbreviations: DFS, double fortified salt; IS, iodized salt; Q_5_FS, quintuple fortified salt; SD, standard deviation.

### Comparison of Sensory Scores

Friedman nonparametric test was used to compare the sensory attributes of Appearance and Color, with mean domain scores following a non-normal distribution. We found no difference in the scores for the Appearance attribute among the 3 salt types (*P* > .05). However, a significant difference was observed in the scoring for the Color attribute (*P* <.05; Table 3). We did a post hoc pairwise-Wilcoxon signed-rank test to compare each pair of salts for the Color attribute. The post hoc comparison indicated that the Color score of IS was significantly higher (*P* < .05), however, there was no difference between the scores of DFS and Q_5_FS. After comparing the mean scores of individual dishes, we find that the pattern is not uniform across the dishes, with some dishes scoring better for Q_5_FS than IS for the sensory attribute of Color (Table 2). Considering the near similar scores for the 3 salt formulations, the statistical difference for the Color attribute between Q_5_FS and IS could be a result of an overfit model rather than any true differences. Future studies replicating the sensory evaluation with a larger sample size may further clarify this assumption.

**Table 3.** Distribution of Sensory Scores for Appearance and Color Between Salts.

		N	Min	Median	**First Q**	Third Q	M**ax**	**Statistic**	***P* value^a^**
Appearance	IS	45	24	39	36	42	47	5.75	.0563
	DFS	45	27	39	36	42	48		
	Q_5_FS	45	32	38	35	41	48		
Color	IS	45	24	40	36	43	47	12.86	.0016^b^
	DFS	45	30	37	35	42	49		
	Q_5_FS	45	31	38	35	41	47		

Abbreviations: DFS, double fortified salt; IS, iodized salt; max, maximum; min, minimum; Q, quartile; Q_5_FS, quintuple fortified salt.

^a^ *P* value from Friedman test for comparison between salts.

^b^ Significantly different from IS at *P* < .05 by Wilcoxon signed-rank test.

We performed a repeated measures ANOVA for the domain scores that were normally distributed, namely aroma, taste, texture, aftertaste, and overall acceptability. Both Q_5_FS and DFS had a slightly lower mean score than IS for almost all the sensory parameters and overall acceptability, but the difference was not significant (Table 4). Therefore, the 3 salts, IS, DFS, and Q_5_FS, were similar to each other in these sensory properties and in overall acceptability.

**Table 4.** Distribution of Sensory Scores for Aroma, Taste, Texture, Aftertaste, and Overall Score Between Salts.

		N	Min	Mean	SD	Max	Statistic	*P* value^a^
Aroma	IS	45	27	39	4.43	49	0.99	.37
	DFS	45	28	38	4.54	50		
	Q_5_FS	45	29	38	4.18	47		
Taste	IS	45	29	38	4.32	46	2.58	.08
	DFS	45	30	38	4.60	49		
	Q_5_FS	45	29	37	4.45	46		
Texture	IS	45	25	39	4.71	48	0.3	.74
	DFS	45	31	39	4.70	48		
	Q_5_FS	45	31	39	4.07	48		
Aftertaste	IS	45	25	39	5.00	48	0.07	.93
	DFS	45	30	39	4.69	47		
	Q_5_FS	45	30	39	4.50	47		
Overall (reported)	IS	45	26	39	4.76	48	0.65	.53
	DFS	45	29	39	4.57	48		
	Q_5_FS	45	31	39	4.21	47		

Abbreviations: ANOVA, analysis of variance; DFS, double fortified salt; IS, iodized salt; max, maximum; min, minimum; Q_5_FS, quintuple fortified salt; SD, standard deviation.

^a^ *P* value from repeated measures ANOVA for comparison between salts.

## Discussion

Iodization of table salt is a proven global health success^14^ and presents an opportunity to build on existing resources for addressing other MNDs. Double fortified salt—IS fortified with iron—has been tested and scaled up in India in the recent past. The University of Toronto formulation for DFS used improved microencapsulation technology to reduce organoleptic barriers to the double fortification of salt. However, some food items were found to have a residual color change in food due to the presence of iron. Research and implementation experience suggest that the color change, though minimal, presents challenges to the acceptability of DFS.^15^ The color change in food cooked with DFS found to be acceptable in ideal conditions (laboratory sensory trials and efficacy studies) may present as a challenge in commercial scale-up when the quality of premix (encapsulation) and/or base salt is compromised.^16^ To that extent, field application of Q_5_FS will need strict and continuous quality monitoring to be successful.

As newer multiple fortified salt formulations are introduced to target multiple MNDs, these formulations must be tested for the acceptability of their sensory properties. In this sensory trial, we have compared the sensory properties of Q_5_FS to that of a high-grade (quality) IS and DFS. However, in real-life situations, consumers would compare the Q_5_FS to that of their regular salt that varies among households, and therefore the consumer acceptability could vary. This sensory trial finds that Q_5_FS scored similarly to DFS and IS in all sensory attributes evaluated, except for color. Our observation of the dishes indicates that (under ideal conditions and with expectations of quality of Q_5_FS met) the color change is mild, and therefore if presenting as a barrier to consumer acceptance, can be overcome with consumer education. The color change in food cooked with Q_5_FS and DFS is primarily due to the presence of iron, a compound having a natural dark brown color. The color change due to iron has been reduced to a large extent by using encapsulated ferrous fumarate premix; however, a change in formulation could be an area of future technological research. There is evidence from the biofortification sector of similar sensory challenges being surpassed with consumer education and behavior change.^17^ Information on the nutritional benefits of fortified foods has been found to increase acceptability and willingness to pay for fortified foods.^17^ Interventions and investments in educating consumers on fortified foods have been minimal, and consumer research in this area has been inadequate. The influence of information and awareness on the acceptability of MFS remains to be evaluated, especially in large-scale trials, and remains an important area of future research.

This sensory trial proves Q_5_FS as an organoleptically acceptable product in ideal laboratory conditions. Since this is an innovative MFS formulation and the study is the first to evaluate its sensory properties, we currently do not have any reference to compare our results with. Also, future research on consumer acceptability studies and the effect of promotional strategies, such as providing information on the nutrient content of Q_5_FS, are required. We conducted a sequential mixed-methods formative study (published separately) to explore the consumer acceptability of quadruple fortified salt among rural low-income populations, which are key target demography for large-scale food-fortification interventions in India. However, large-scale trials are suggested for future research.

## Conclusion

This sensory evaluation successfully demonstrates that, except for the Color attribute, there are no significant differences in the sensory attributes of the IS, DFS, and Q_5_FS. The Q_5_FS score equally well on the overall acceptability parameter as DFS and IS. This study concludes that Q_5_FS is organoleptically acceptable under ideal conditions. However, these properties need to be tested in real-world conditions. Tackling the long-standing public health challenge of MNDs needs innovative strategies, and MFS shows promising potential.
